# Anharmonic Effects on the Thermodynamic Properties of Quartz from First Principles Calculations

**DOI:** 10.3390/e23101366

**Published:** 2021-10-19

**Authors:** Mara Murri, Mauro Prencipe

**Affiliations:** 1 Department of Earth and Environmental Sciences, University of Milano-Bicocca, Piazza della Scienza 4, I-20126 Milano, Italy; 2 Earth Sciences Department, University of Torino, Via Valperga Caluso 35, I-10125 Torino, Italy; mauro.prencipe@unito.it

**Keywords:** quartz, anharmonicity, phonon dispersion, phase transition, bulk modulus

## Abstract

The simple chemistry and structure of quartz together with its abundance in nature and its piezoelectric properties make convenient its employment for several applications, from engineering to Earth sciences. For these purposes, the quartz equations of state, thermoelastic and thermodynamic properties have been studied since decades. Alpha quartz is stable up to 2.5 GPa at room temperature where it converts to coesite, and at ambient pressure up to 847 K where it transforms to the beta phase. In particular, the displacive phase transition at 847 K at ambient pressure is driven by intrinsic anharmonicity effects (soft-mode phase transition) and its precise mechanism is difficult to be investigated experimentally. Therefore, we studied these anharmonic effects by means of ab initio calculations in the framework of the statistical thermodynamics approach. We determined the principal thermodynamic quantities accounting for the intrinsic anharmonicity and compared them against experimental data. Our results up to 700 K show a very good agreement with experiments. The same procedures and algorithms illustrated here can also be applied to determine the thermodynamic properties of other crystalline phases possibly affected by intrinsic anharmonic effects, that could partially invalidate the standard quasi-harmonic approach.

## 1. Introduction

Alpha quartz is a very common mineral in everyday life. Indeed, its piezoelectric properties make it suitable for engineering applications as oscillator and sensor in electronic devices (e.g., [1,2]) and it is also one of the main constituents in the Earth’s crust. Quartz usually occurs as a free crystal in magmatic and sedimentary rocks and as an inclusion phase entrapped in other minerals (i.e., host-inclusion systems) in metamorphic rocks.

Moreover, since the abundance of quartz in a variety of geological environments and its multiple applications in material sciences, high-pressure and high-temperature experiments have been carried out to determine and constrain its thermoelastic properties (e.g., [3,4,5,6,7,8]). Furthermore, it has also been recently studied in the framework of Raman elastic geobarometry applications (e.g., [9,10,11,12,13]) to calculate the entrapment conditions (i.e., pressure and temperature) of host-inclusion systems found in metamorphic rocks (e.g., [14,15,16]).

Quartz, despite having a simple chemistry (i.e., SiO_2_) and structure (framework of SiO_4_ tetrahedra), has several high-pressure and high-temperature polymorphs. In particular, in temperature (at ambient pressure) quartz shows a displacive phase transition at about 847 K [3] from the α phase (trigonal) to the β phase (hexagonal) which makes quite difficult to model its behavior as a function of temperature through and above the phase transition itself, whereas in pressure (at room temperature) it is stable up to 2.5 GPa (quartz-coesite phase transition, see [5,17]). Recently, reliable pressure-volume-temperature equations of state (PVT EoS) for quartz have been determined accounting for the continuous phase transition in temperature [8]. A reliable determination of the bulk modulus (K) as a function of temperature has been determined from the PVT experimental data, directly using its definition (involving the partial derivative of the pressure with respect to the volume, at any given temperature) instead of some EoS fit, due to its strong correlation with the K’ parameter (essentially, the first derivative of K with respect to P in the expression K = K_0_ + K’P) and the significant dependence of K’ on temperature (e.g., [8]).

Behind the α → β phase transition there are effects played by *intrinsic anharmonicity* which cannot be directly studied experimentally. Therefore, in this study we evaluate for the first time the role and the effects of the anharmonicity on the thermoelastic properties of quartz in the α stability field and across the α → β phase transition by means of first principles simulations in the framework of the statistical thermodynamics approach. The ab initio results are then compared against the experimental data and the effects of the anharmonicity on the estimation of the transition temperature are also discussed.

Moreover, we also provide the guidelines and the tools to apply this study on other mineral phases.

## 2. Methods and Computational Details

### 2.1. Strategy of Computation

Apart from the correction for intrinsic anharmonicity involving a number of vibrational modes, the calculations are performed assuming the quasi-harmonic approximation (QHA): vibrational frequencies are computed in the harmonic approximation for each value of the unit-cell volume in a given range (see [18,19] for detailed explanations). Within the framework of statistical thermodynamics, for each value of the unit-cell volume, frequencies together with their volume dependence and the values of the static energies (energies at *clamped* nuclei), are used to compute the partition function and all the quantities related to it: the Helmholtz free energy function *F* (*V*,*T*) and the pressure at any given volume and temperature from the definition:

(1) $$P=-{\left(\frac{\partial F}{\partial V}\right)}_{T}$$

Phonon dispersion has been accounted for by performing the frequency calculations for supercells (of the primitive cell of α-quartz) to sample several points of the Brillouin zone.

As the intrinsic anharmonicity is concerned, the shape of the potential energy function (*E*) as the nuclei are displaced along each normal mode coordinate (Q) was checked for low frequency modes and, in particular, for the soft-mode driving the α → β transition. Significant deviations of the *E* (Q) functions from the quadratic behavior directly reveal the failure of the harmonic approximation for the investigated normal modes. In these cases, for each anharmonic normal mode, the *E* (Q) function was fitted by a fourth order polynomial of Q, and the vibrational energy levels were computed following a standard *variational scheme* in which (*i*) the resulting anharmonic Hamiltonian of the oscillator is represented in the basis of the harmonic functions and (*ii*) the resulting Hamiltonian matrix is diagonalized, where each eigenvalue represents a particular vibrational energy level. Such a procedure was repeated for different values of the unit-cell volume. As the energy levels of the anharmonic oscillator are generally *not equally spaced*, the partition function corresponding to each anharmonic oscillator was directly summed term by term until convergence was reached. For each anharmonic oscillator, the Helmholtz *F* (*V*,*T*) function was then computed in given *V*,*T* ranges and added to the *standard* contribution from the harmonic modes to obtain the total Helmholtz free energy as a function of volume and temperature.

### 2.2. Computational Details

**Figure 1:**
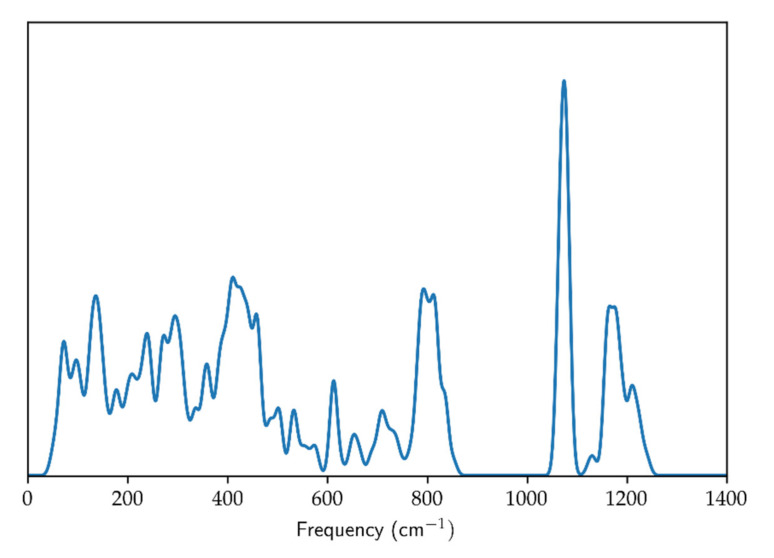
Quartz phonon density of state.

Optimized geometry (cell parameters and fractional coordinates), energy and frequencies of vibrational modes were computed by means of the CRYSTAL17 code [20]. The WC1LYP hybrid Hartree-Fock/Density Functional Theory (HF/DFT) functional for the electronic exchange and correlation was chosen; precisely the Wu-Cohen [21] GGA functional for the exchange part, mixed with 16% of *non-local* exact Hartree-Fock exchange, and the LYP functional [22] for the correlation part. The WC1LYP formulation proved to be particularly effective in the reliable reproduction of geometry, elastic properties and vibrational frequencies of silicates (see [19]). The localized basis sets chosen were 86-311d1G for Si [23] and 84-11d11G for O [24] (already used for the successful computation of vibrational frequencies of other silicates: e.g., [25]). The computation was repeated at 12 different values of the primitive unit-cell volume, in the 93–111 Å^3^ volume range. Phonon dispersion correction was accounted for by computing the vibrational frequencies with the supercell approach: the 2 × 2 × 2, 3 × 3 × 3, 4 × 1 × 1 (and the symmetry equivalent 1 × 4 × 1 supercell) and 1 × 1 × 4 supercells were employed; the resulting phonon density of state (PDOS) is shown in Figure 1.

The processing of the ab initio data obtained by means of the CRYSTAL17 program was performed by the QM-thermodynamic program [26].

The anharmonic correction was performed for the three modes in the lowest frequency range: the A_1_ vibrational mode (the soft-mode), the E mode and the mode of symmetry (2) at the K-point (0 0 1/2) in the Brillouin zone. The E (Q) energy profile along each anharmonic mode was computed by means of the SCANMODE keyword in the CRYSTAL17 input file [20]. Energy data from the SCANMODE’S were processed by means of the *anharm* module of the QM-thermodynamic program [26]. Some details of the implementation are provided in the form of a tutorial on the program website (https://qm-thermodynamics.readthedocs.io/en/main/_static/anharm.html (accessed on 1 September 2021).

It is to be noted that the procedure followed in the present work is not equivalent to the one implemented in the CRYSTAL17 code; indeed, the latter is tailored to estimate the anharmonic correction to the vibrational frequencies of possible X-H stretching modes, by shifting the position of a given H nucleus along the direction of the X-H bond, and not along the direction of the corresponding normal mode.

## 3. Results and Discussion

The isothermal Reuss bulk modulus (which measures the volume response of a material to hydrostatic pressure) of quartz has been calculated as follows:

(2) $$K=-V{\left(\frac{dP}{dV}\right)}_{T}$$

**Figure 2:**
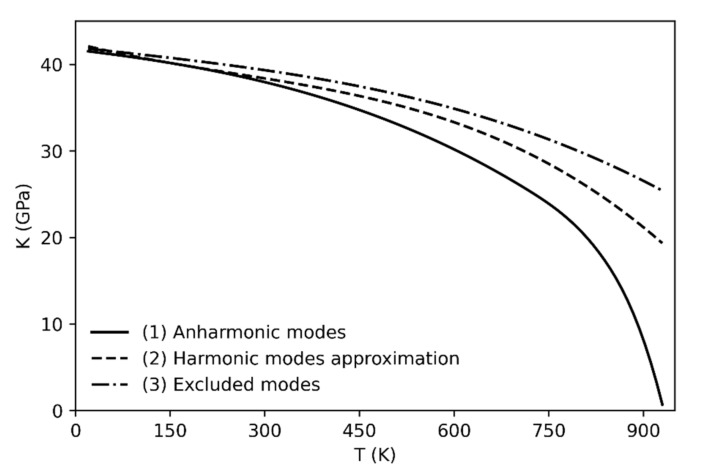
Calculated bulk modulus including the anharmonic correction (1), within the harmonic approximation (2) and excluding the anharmonic modes (3).

The determination of the bulk modulus by using Equation (2) has been carried out in three ways (always accounting for the phonon dispersion): (1) by including the anharmonicity correction, (2) within the harmonic approximation and (3) by excluding the three anharmonic modes (see Figure 2). The anharmonic correction (1) reproduces the experimental trend of the bulk modulus as a function of temperature [8]. Indeed, the bulk modulus values drop towards zero as the phase transition is approached.

On the other hand, when the anharmonicity is not taken into account either by using the harmonic approximation or by excluding the anharmonic modes from the calculation (cases 2 and 3), the bulk modulus decreases much more slowly with respect to the trend obtained in case (1), and with respect to the experiment [8]; therefore the α → β quartz phase transition is not predicted (or it is predicted at significantly higher temperature than the experimental observation). The worse results are obtained when the anharmonic modes are not taken into account (see Figure 2, line 3) since the trend still shows high bulk modulus values at temperatures higher than 1000 K.

**Figure 3:**
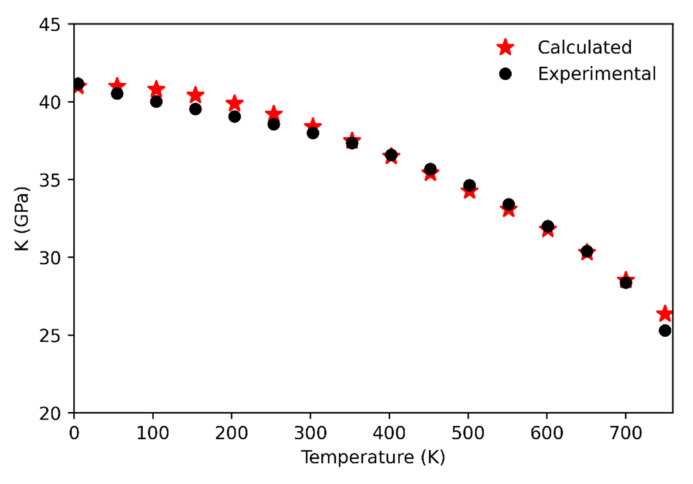
Calculated (this study) and experimental [8] quartz bulk modulus up to 750 K.

Concerning the comparison with the experiments, the calculated bulk modulus values up to 750 K have been compared against the data by [8] (see Figure 3). The agreement between computational and experimental data is very good, with the highest discrepancy being of about 0.7 GPa at 750 K. At higher temperatures the discrepancy progressively increases, probably due to an underestimation of the computed thermal expansion (see below). Indeed, the phase transition to the β phase is estimated to occur at a higher temperature (~990 K; that is the temperature at which the bulk modulus falls to zero and the unit-cell volume reaches the point where the frequency of the soft-mode shows its minimum) with respect to the experimental data (~847 K). The role of anharmonicity appears to be quite negligible up to 750 K showing a small impact on the behavior of the bulk modulus. On the contrary, the anharmonicity is the key for the correct reproduction of the bulk modulus behavior beyond 750 K, even if the phase transition is predicted to occur at a higher temperature than the experimental one. Indeed, in the high temperature range, that computed quantity is generally overestimated with respect to the experimental datum at the corresponding temperature.

**Figure 4:**
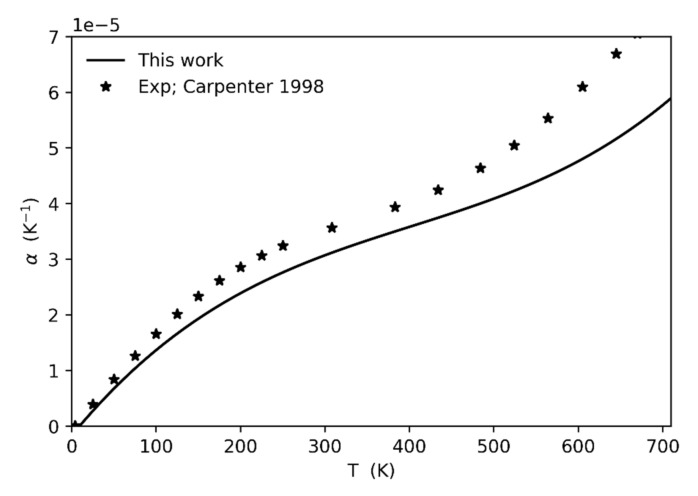
Comparison between the calculated thermal expansion values (this study) and those derived from the experimental data by [3].

In order to investigate the reason of such an overestimation of the bulk modulus as the transition temperature is approached, we computed the thermal expansion values up to 700 K and compared it to the values derived from the experiments [3]. The agreement between calculated and experimental data is quite good up to 500 K, then it becomes worse at higher temperatures (see Figure 4). In particular, the calculated thermal expansion is generally underestimated with respect to the experiments, with a discrepancy increasing as the temperature increases. Such underestimation is probably due to several factors, the main one being related to the type of correction adopted for anharmonicity that does not include possible, as well as probable, phonon-phonon interactions. Intrinsic anharmonicity is therefore not fully corrected, leading to an underestimation of the phonon pressure and hence of the thermal expansion. As a consequence, the unit-cell volume at which the frequency of the soft-mode falls to zero (at least if calculated in the harmonic approximation) is associated to a temperature that is higher (of about 140 K) than the experimental one at which the phase transition is really observed.

**Figure 5:**
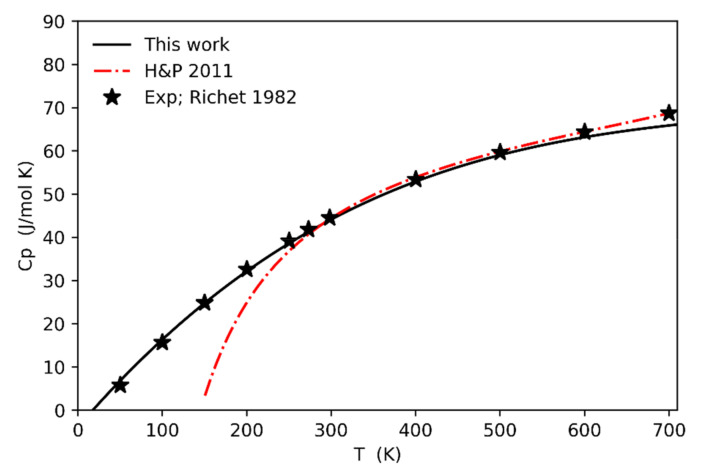
Calculated specific heat (C_p_) as a function of temperature at constant pressure (solid line) and comparison against the experimental data from [27] and those derived from the thermodynamic database by [5].

Similar considerations can be performed for other thermodynamic quantities such as, for instance, the specific heat at constant pressure (Figure 5; solid line).

The comparison of the computed data (including the anharmonic correction) against experimental ones is very good from room temperature (i.e., 300 K) to high-temperature values up to 700 K, then we start to underestimate the specific heat. This underestimation could be related to the behavior observed in the thermal expansion (Figure 4) at high temperatures (above 500 K) since C_p_ = C_v_ + *VTK*α^2^. On the other hand, at low temperatures (from 300 K down to 0 K) the computed specific heat perfectly follows the trend of the experimental data by [27] (the curve from [5] is the result of a power series fitting of experimental data from the high temperature region down to 300 K, whereas those below 300 K have been extrapolated by the same power series, and therefore they should not be considered).

**Figure 6:**
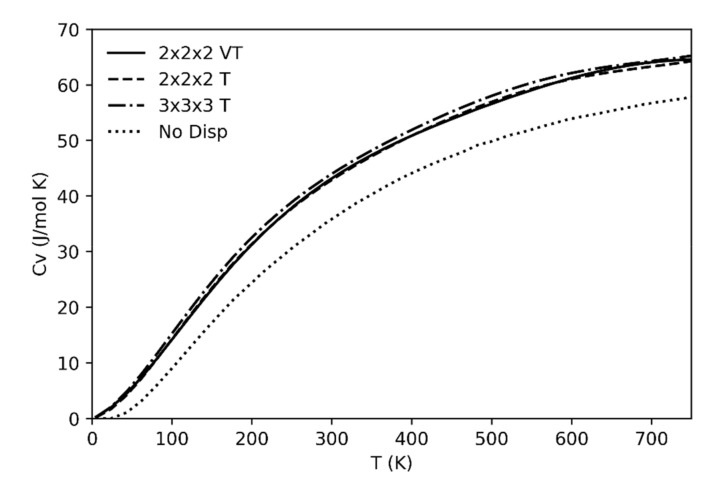
Calculated specific heat (C_v_) as a function of temperature and a constant volume. The solid line is the C_v_ calculated including the phonon dispersion in a supercell 2 × 2 × 2 with the calculated frequencies over a range of unit-cell volumes; the dashed line reports the C_v_ obtained from a calculation of a supercell 2 × 2 × 2; dotted-dashed line shows the C_v_ calculated from a calculation of a supercell 3 × 3 × 3; the dotted line is the C_v_ calculated including only the vibrational modes at the Brillouin zone center.

The specific heat as a function of temperature at constant volume (Cv) has also been calculated (Figure 6). As already discussed for the previous thermodynamic quantities, if we leave out the anharmonicity effects the changes will be negligible in the considered range of temperatures far from the phase transition (i.e., up to 700 K).

As phonon dispersion effects are concerned, the importance of including in the calculation set of frequencies of modes off the Brillouin zone center and, in particular, of considering the contribution from the acoustic branches (absent if only the frequencies at the center of the Brillouin zone are considered) is also well illustrated in Figure 6. Four different cases are reported referring to computations not including acoustic branches and optical phonon dispersion (dotted line) or including an increasingly higher number of frequencies from supercell calculations. The neglection of acoustic branches results of a significant underestimation of Cv; on the other hand, no supercells larger than 2 × 2 × 2 are required to reach convergence in the results. At variance with the other cases, the continuous line refers to the case where the supercell frequencies are computed at different volumes of the unit cell (in the spirit of the quasi-harmonic approximation); this latter procedure has essentially no effect on the resulting Cv curve.

**Figure 7:**
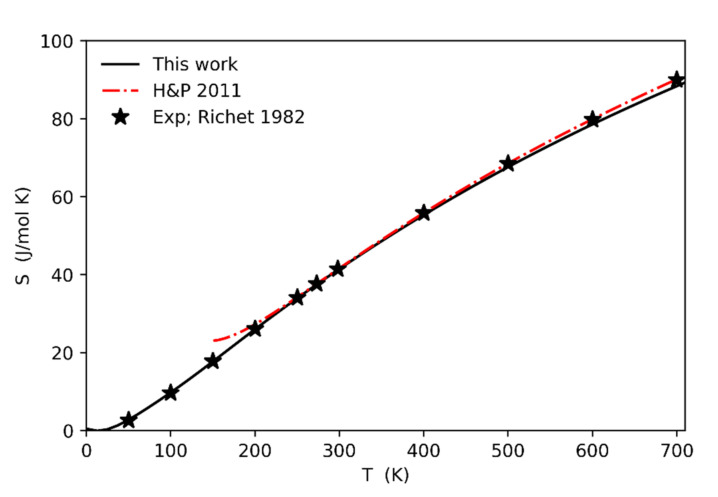
Calculated entropy values as a function of temperature (solid line) and comparison against the experimental data from [27] and those derived from the thermodynamic database by [5].

Figure 7 reports the comparison between the calculated entropy values (solid line) against the experimental ones from [5,27]. The agreement is very good in the whole range of temperatures (0–700 K).

**Figure 8:**
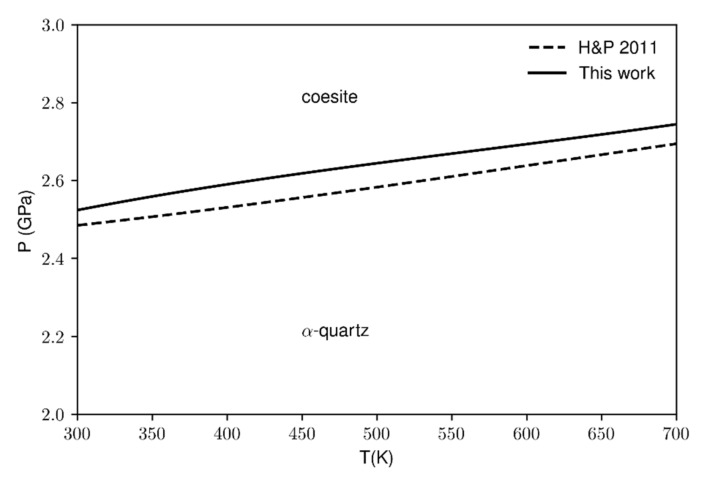
Quartz-coesite phase boundary. Comparison between the calculated data (solid line) and the experimental ones from the thermodynamic database by [5].

The computed values of the specific heat, entropy, bulk modulus and thermal expansion have been used to calculate the quartz-coesite phase boundary. Our data (Figure 8 solid line) have been compared against the experimental data from the thermodynamic database by [5]. The temperature ranges from 300 to 700 K due to the limited availability of literature data and also because the calculated phase boundary at higher temperatures (>700 K) would be affected by the soft-mode. However, as reported in Figure 8, the agreement between the calculated and experimental values is very good in the considered temperature range. Moreover, although the calculations slightly overestimate the transition pressures in the range of the selected temperatures, the Clapeyron slope for both the lines is the same within the second decimal digit (5.32 bar/K) which means that the two-phase boundaries are parallel one to another.

## 4. Conclusions

We have computed for the first time the main thermodynamic quantities of α-quartz by means of ab initio CRYSTAL17 software coupled with the QM-thermodynamic program [26] to account for intrinsic anharmonic effects. The calculations have shown that the intrinsic anharmonicity plays a fundamental role at temperatures close to the phase transition, whereas at lower temperatures (i.e., from 0 K up to 700 K) it does not significantly affect the behavior of the computed thermodynamic quantities. Indeed, all our calculations performed up to 700 K are in very good agreement with the experimental data. Therefore, the ab initio simulations coupled with the data processing using the QM-thermodynamic program are reliable and, thus, can also be performed for other crystalline phases to explore those effects (e.g., intrinsic anharmonicity) which cannot be directly studied experimentally.

The QM-thermodynamic program is written in Python language. The program is free for non-commercial use and available for download from Github (http://qm-thermodynamics.readthedocs.io/ (accessed on 2 July 2021)) together with detailed documentations (e.g., input description, explanations and tutorials). The program can be loaded and run in a normal Python console as well as in any other environment such as Spyder (https://www.spyder-ide.org/ (accessed on 2 July 2021)) or even in a browser by means of Jupyter notebooks (https://jupyter.org/ (accessed on 2 July 2021)).

## Data Availability

Not applicable.
